# Comparison of Pulse Palpation and Paramedic-Performed Doppler Ultrasonography for Assessing Distal Circulation After Tourniquet Application: A Randomized Crossover Study

**DOI:** 10.3390/healthcare14203367

**Published:** 2026-10-09

**Authors:** Suil Kim, Eun-Ji Yoo, Hyeong-Tae Kim, Yun-Deok Jang

**Affiliations:** 1 Department of Paramedicine, Yeungjin University, Daegu 41527, Republic of Korea; avantetop@hanmail.net; 2 United States Army Garrison (USAG) Humphreys, Pyeongtaek 96271, Republic of Korea; angieunji@gmail.com; 3 SL Emergency Medical Mobility Research Institute; Wonju 26322, Republic of Korea; kkkhht@hanmail.net; 4 Department of Paramedicine, Tongmyong University, Busan 48520, Republic of Korea

**Keywords:** tourniquet, Doppler ultrasonography, pulse palpation, hemorrhage control, paramedics, prehospital emergency care, peripheral circulation

## Abstract

**Background/Objectives:** Accurate assessment of distal circulation is essential for confirming effective arterial occlusion after tourniquet application in patients with life-threatening extremity hemorrhage. Although distal pulse palpation is commonly used in prehospital settings, its accuracy may be affected by examiner experience and environmental conditions. This study aimed to compare manual pulse palpation and paramedic-performed Doppler ultrasonography for assessing distal circulation after tourniquet application, using assessor-performed Doppler ultrasonography as the reference assessment. **Methods:** A prospective randomized crossover study was conducted among 41 actively practicing paramedics. Participants were randomly assigned to one of two assessment sequences: pulse palpation followed by Doppler ultrasonography or Doppler ultrasonography followed by pulse palpation. After standardized tourniquet application to the upper extremity, distal circulation was independently assessed using both methods. Assessor-performed Doppler ultrasonography served as the reference assessment. Sensitivity, specificity, accuracy, positive predictive value (PPV), and negative predictive value (NPV) were calculated for each method. Agreement between the two index tests was evaluated using percent agreement and Cohen’s kappa, and assessment times were compared between methods. **Results:** Among the 41 participants, the reference standard identified distal flow in 18 (43.9%) and absence of distal flow in 23 (56.1%). Relative to the assessor-performed Doppler reference assessment, pulse palpation demonstrated a sensitivity of 66.7% (95% CI, 41.0–86.7), specificity of 87.0% (95% CI, 66.4–97.2), and accuracy of 78.0% (95% CI, 62.4–89.4). In comparison, paramedic-performed Doppler ultrasonography demonstrated a sensitivity of 88.9% (95% CI, 65.3–98.6), specificity of 91.3% (95% CI, 72.0–98.9), and accuracy of 90.2% (95% CI, 76.9–97.3). The PPV and NPV were 80.0% and 76.9% for pulse palpation and 88.9% and 91.3% for Doppler ultrasonography, respectively. Agreement between the two methods was 82.9%, with a Cohen’s kappa of 0.65. Mean assessment time was 14.2 ± 6.1 s for pulse palpation and 28.5 ± 10.4 s for Doppler ultrasonography. **Conclusions:** Paramedic-performed Doppler ultrasonography showed greater concordance with assessor-performed Doppler ultrasonography than pulse palpation for assessing distal circulation after tourniquet application, although it required additional assessment time. Because assessor-performed Doppler ultrasonography served as the reference assessment rather than an independent physiological gold standard, these findings should be interpreted as reflecting agreement with the reference Doppler assessment rather than definitive diagnostic superiority. Handheld Doppler ultrasonography may therefore have a potential role as an adjunct to pulse palpation, particularly when palpation findings are equivocal or objective reassessment of distal flow is required. Further studies using independent reference standards and conducted in real-world prehospital environments are warranted to evaluate its clinical utility, feasibility, and safety.

## 1. Introduction

Trauma (injury) is a major cause of death and one of the most important global public health problems. According to the World Health Organization (WHO), approximately 4.4 million people die from injuries each year, accounting for about 8% of all deaths worldwide [1]. These deaths include a wide range of causes such as road traffic crashes, falls, drowning, burns, poisoning, and violence, and injuries are also a leading cause of death, particularly among younger age groups [1].

Previous studies have reported that hemorrhage accounts for approximately 30–40% of all trauma-related deaths, and that 33–56% of these hemorrhagic deaths occur in the prehospital phase [2]. Thus, hemorrhage is a time-critical problem, and rapid recognition and bleeding control in the prehospital setting are key determinants of patient outcomes [2].

This body of evidence has led to changes in initial trauma assessment systems. In the recently revised Advanced Trauma Life Support (ATLS) 11th edition, the primary survey algorithm has been re-established as xABCDE, emphasizing immediate control of exsanguinating external hemorrhage (the “eXsanguinating”) as the highest priority [3].

In particular, tourniquet application is widely recommended as a core intervention for life-threatening extremity hemorrhage in the prehospital setting, and its use has rapidly expanded in civilian environments as well [4]. Prior studies have reported that prehospital tourniquet use for extremity vascular injuries is associated with reduced mortality, and that preventing ongoing blood loss through reassessment during transport can positively influence survival [5,6]. In this context, not only “whether a tourniquet is used,” but also appropriate application and continuous reassessment are known to affect hemostatic effectiveness and survival.

Current trauma and hemorrhage-control guidance emphasizes reassessment of tourniquet effectiveness after application, including confirmation of hemorrhage control and assessment for persistent distal arterial flow [7,8,9,10]. However, distal pulse palpation is subjective, and its reliability may vary depending on the surrounding environment and the examiner’s experience. Indeed, in “busy clinical settings,” peripheral pulse palpation has been associated with reduced inter-examiner agreement and accuracy errors, suggesting limitations in using palpation alone to evaluate flow control [11]. Therefore, there is a need for more objective and reproducible tools for post-tourniquet assessment. Notably, research on diverse methods for assessing blood flow after tourniquet application remains scarce.

Accordingly, we propose the use of handheld Doppler ultrasound as a new alternative for evaluating blood flow occlusion after tourniquet application. Doppler ultrasound enables rapid and objective assessment of blood flow and is widely used in clinical practice; moreover, it can be applied to patients as a noninvasive method.

Therefore, the purpose of this study was to compare manual pulse palpation and paramedic-performed Doppler ultrasonography for assessing distal circulation after tourniquet application, using assessor-performed Doppler ultrasonography as the reference assessment, and to evaluate the agreement of each method with the reference assessment.

## 2. Materials and Methods

### 2.1. Study Design

This study employed a prospective randomized crossover design to compare, within the same participants, the accuracy of two methods for assessing distal circulation after tourniquet application: pulse palpation and Doppler ultrasonography. The study was conducted from January 2026 to August 2026. All participants received a full explanation of the study purpose and procedures and provided written informed consent prior to participation. The study was conducted after approval by the Institutional Review Board (IRB No. 2026-01-019).

### 2.2. Study Population

Participants were actively practicing paramedics recruited nationwide, and the study was conducted among National Fire Agency 119 emergency medical technicians who participated in the prehospital trauma life support (PHTLS) provider course delivered by NAEMT Education Korea. A total of 45 individuals were assessed for eligibility. Eligible participants were licensed paramedics who were currently engaged in prehospital emergency care and who had prior exposure to tourniquet use, defined as having completed at least one tourniquet training session and/or having previously applied a tourniquet to a patient in the field, and who voluntarily agreed to participate after providing written informed consent. Individuals were excluded if they were not currently working in prehospital emergency care, had no prior tourniquet training or practical experience, had upper or lower extremity trauma, fractures, vascular disease, or any other condition that could affect peripheral circulation, were pregnant or had given birth within the previous three months, or were unable to complete study procedures because they withdrew consent or did not sufficiently understand the protocol. Of the 45 individuals screened, four were excluded: two did not meet the inclusion criteria, one declined to participate, and one was excluded for other reasons. As a result, 41 participants were randomized, with 21 assigned to Sequence A in which pulse palpation was performed first followed by Doppler ultrasonography, and 20 assigned to Sequence B in which Doppler ultrasonography was performed first followed by pulse palpation. All randomized participants completed both study periods and were included in the final analysis, yielding complete paired data for both methods from all 41 participants.

**Figure 1:**
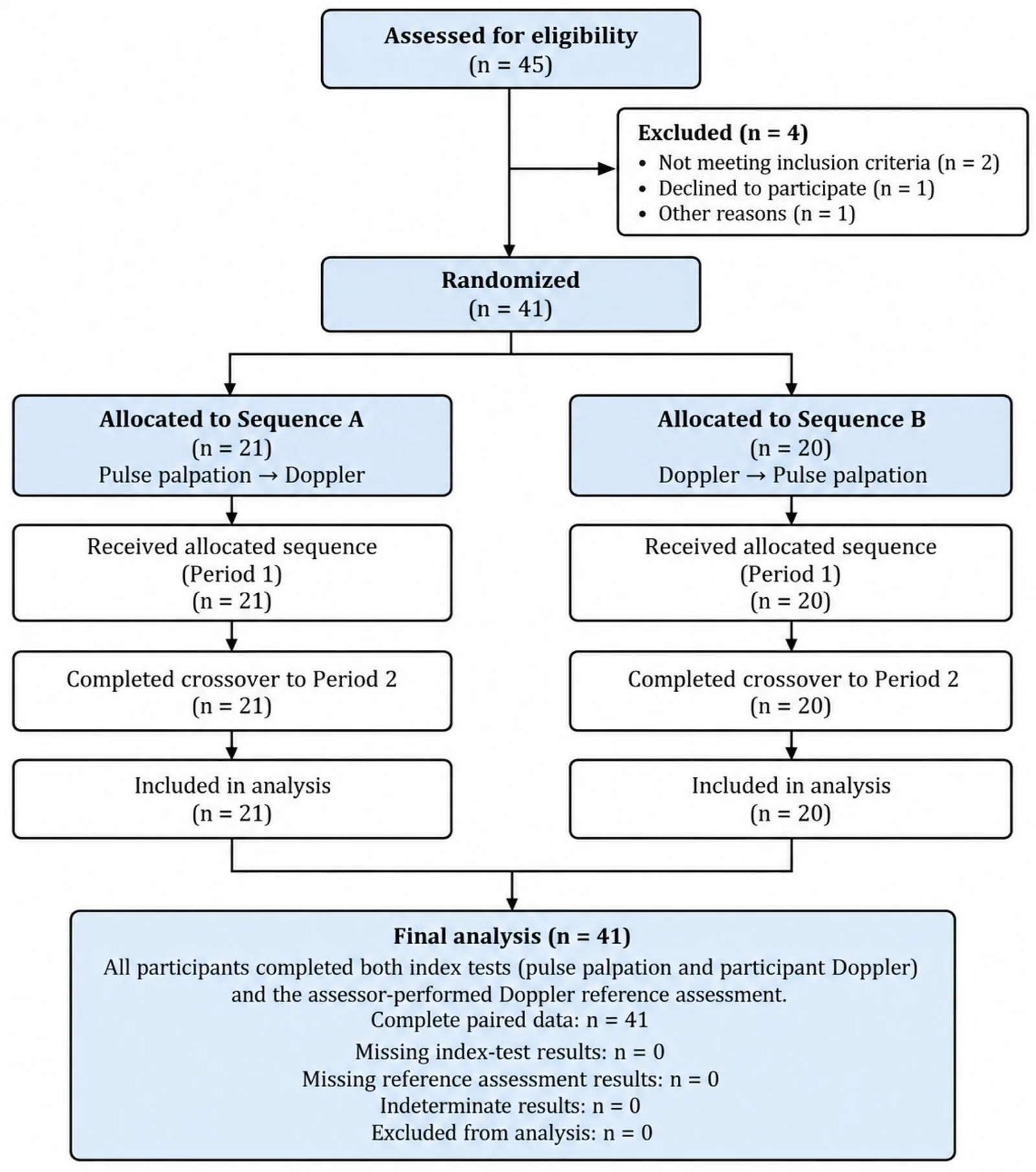
Diagnostic-study participant flow diagram showing enrollment, randomized assessment sequence, completion of the index tests and reference assessment, and inclusion in the final paired analysis.

The sample size was calculated using a formula for paired comparisons in a crossover design. The required sample size was determined a priori using a formula-based calculation for paired comparisons, considering the within-participant comparison inherent in the crossover design. The calculation was based on a two-sided significance level (α) of 0.05, a statistical power (1 − β) of 0.80, and a standardized effect size of 0.50. Based on these assumptions, the minimum required sample size was calculated to be 34 participants. To account for an anticipated dropout rate of approximately 10%, the target sample size was increased to approximately 40 participants. A total of 45 participants were screened for eligibility, of whom 41 were ultimately enrolled, completed both assessment methods, and were included in the final analysis. Ultimately, 41 participants were enrolled, randomized, and analyzed as illustrated in Figure 1.

### 2.3. Study Protocol

This study was designed as a randomized crossover trial to compare the accuracy of manual pulse palpation and Doppler ultrasonography for assessing distal circulation in the upper extremity after tourniquet application. The two index tests were manual pulse palpation and participant-performed Doppler ultrasonography, whereas assessor-performed Doppler ultrasonography served as the reference assessment. Participants consisted of actively practicing National Fire Agency 119 emergency medical technicians working in prehospital settings, and recruitment targeted individuals who had previously received formal tourniquet education and hands-on training. Eligible participants were recruited from trainees attending a prehospital trauma care course and enrolled voluntarily after providing written informed consent.

Before data collection, all participants received a full explanation of the study aims, procedures, and ethical considerations and provided written informed consent. All participants were licensed Level 1 Emergency Medical Technicians actively engaged in prehospital emergency care. None had received prior formal ultrasound training or had previous experience using handheld Doppler ultrasonography. Therefore, before data collection, all participants received a standardized 15-min pre-study training session. The training covered basic operation of the handheld Doppler device, identification of the target artery, probe positioning, acquisition and interpretation of Doppler flow signals, and classification of distal flow as present or absent. Participants were allowed to familiarize themselves with the device during the training session before performing the study assessments. The Doppler component included basic device operation, identification of the target distal artery, probe positioning, acquisition and interpretation of the Doppler flow signal, and binary classification of distal flow as present or absent. Participants were allowed to familiarize themselves with the handheld device during the training session before beginning the study assessments. Participants were then randomly assigned using a computer-generated randomization scheme to one of two sequences: Group A performed pulse palpation first followed by Doppler ultrasonography, whereas Group B performed Doppler ultrasonography first followed by pulse palpation.

The tourniquet was applied to the upper extremity of each study participant according to a standardized application protocol and maintained for 2 min before the assessment began. Distal circulation was assessed at a predefined site over the brachial artery distal to the tourniquet. The same target artery and anatomical site were used for pulse palpation, participant-performed Doppler ultrasonography, and assessor-performed Doppler ultrasonography to ensure consistency across assessments. Each participant assessed distal blood flow using the first randomly assigned method and recorded the presence or absence of distal flow as a binary outcome (Yes/No). After a 1-min interval, the participant repeated the assessment using the second assigned method and again recorded the result as Yes or No.

For Doppler assessment, the brachial artery was first identified at the predefined site distal to the tourniquet using B-mode imaging, followed by pulsed-wave (PW) Doppler assessment of arterial blood flow. Distal flow was predefined as “present” when a reproducible arterial Doppler flow signal was identified at the target site and as “absent” when no reproducible arterial Doppler flow signal was detected at the predefined site. The same Doppler assessment procedure and criteria were applied to both participant-performed and assessor-performed Doppler assessments.

**Figure 2:**
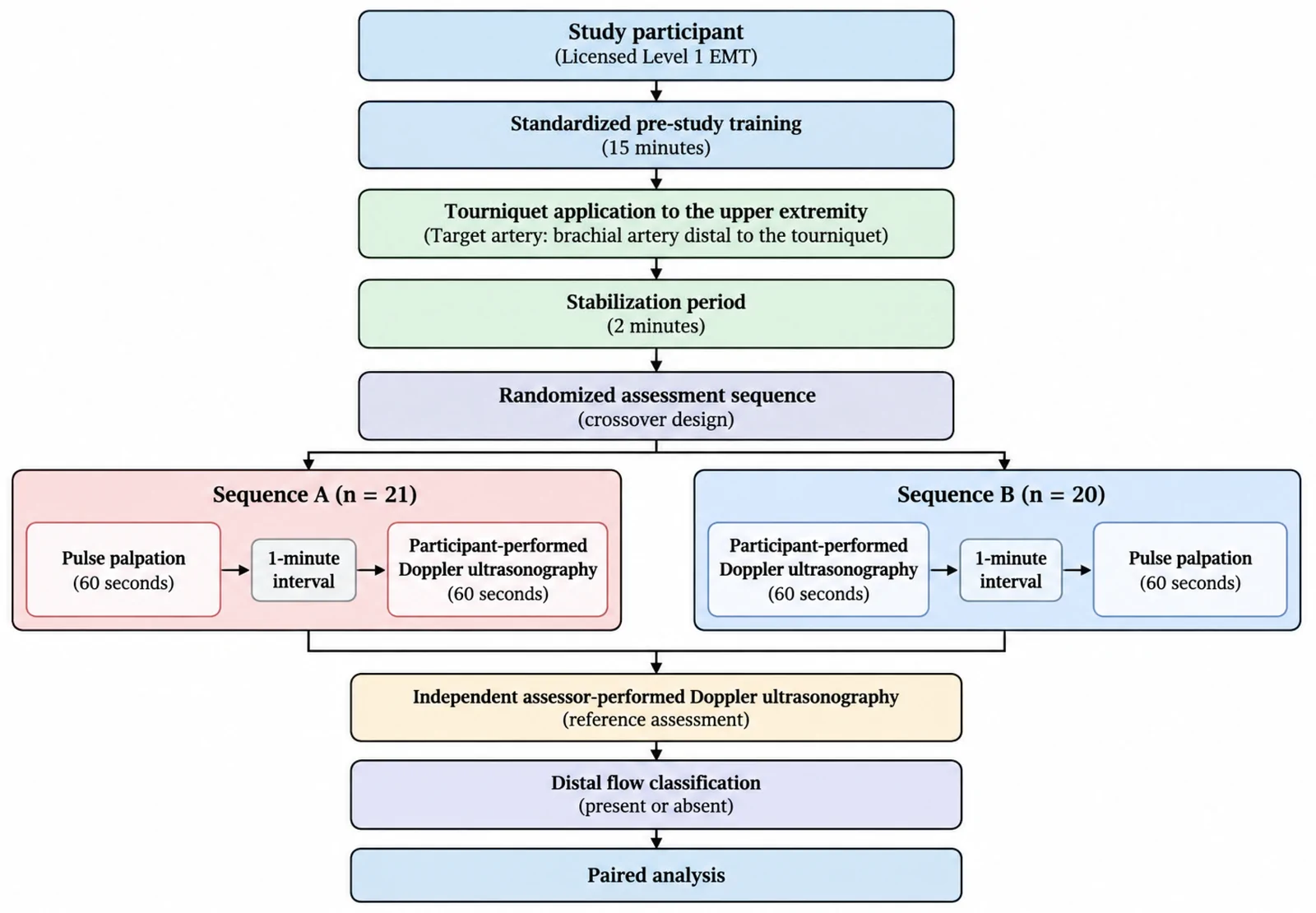
Flow diagram of the randomized crossover assessment protocol.

Assessor-performed Doppler ultrasonography was used as the reference assessment. The reference assessment was performed by an independent assessor who was a professor in the Department of Paramedicine with 20 years of clinical experience and extensive experience in Doppler ultrasonography and ultrasound assessment. The assessor was not involved in the participants’ index-test assessments or recording process and was blinded to the participants’ pulse palpation and participant-performed Doppler results as well as to the randomized assessment sequence. The assessor-performed Doppler assessment was conducted immediately after completion of both index tests while the tourniquet remained in place. The same predefined brachial artery site, Doppler protocol, and flow-classification criteria used for the participant-performed Doppler assessment were applied to the reference assessment. The assessor independently classified distal arterial flow as present or absent without access to the participants’ index-test results. Data collected included participant demographic and professional characteristics, including sex, age, affiliated organization, years of field experience, and prior tourniquet training and practical experience, as well as procedure-related variables, including the assigned assessment sequence and the time required for each assessment. Binary outcomes were recorded for pulse palpation, participant-performed Doppler ultrasonography, and assessor-performed Doppler ultrasonography, with all results documented as Yes or No (Figure 2).

### 2.4. Study Materials

#### 2.4.1. Tourniquet

In this study, the tourniquet used was the combat application tourniquet (CAT), a widely adopted standard device in prehospital trauma care. The CAT is a mechanical windlass tourniquet composed of a strap-and-buckle system and a windlass rod that enables stepwise tightening and fine control of compression. It functions by creating initial tension through strap tightening and then increasing circumferential pressure by rotating the windlass to restrict arterial inflow. Once adequate compression is achieved, the windlass is secured in a retaining clip to minimize loosening during application.

Tourniquet application was performed according to a standardized protocol. The CAT was placed on the participant’s upper extremity, the strap was routed through the buckle, and the strap was pulled firmly to ensure close contact with the skin. The windlass was then rotated to progressively increase pressure until distal perfusion was reduced or absent. After tightening, the windlass was locked into the retaining clip to maintain the target level of compression for the predefined duration specified in the study procedure.

To ensure consistency across participants, the same type of CAT was used throughout the study, and the application site and tightening technique followed the criteria taught during the pre-study training. Immediately after completion of the assessments, the tourniquet was released to avoid unnecessary prolonged ischemia. Participant safety was prioritized, and the procedure was designed to allow immediate discontinuation if concerning symptoms occurred, such as severe pain, numbness, pallor, or other abnormal sensations.

#### 2.4.2. Doppler

**Figure 3:**
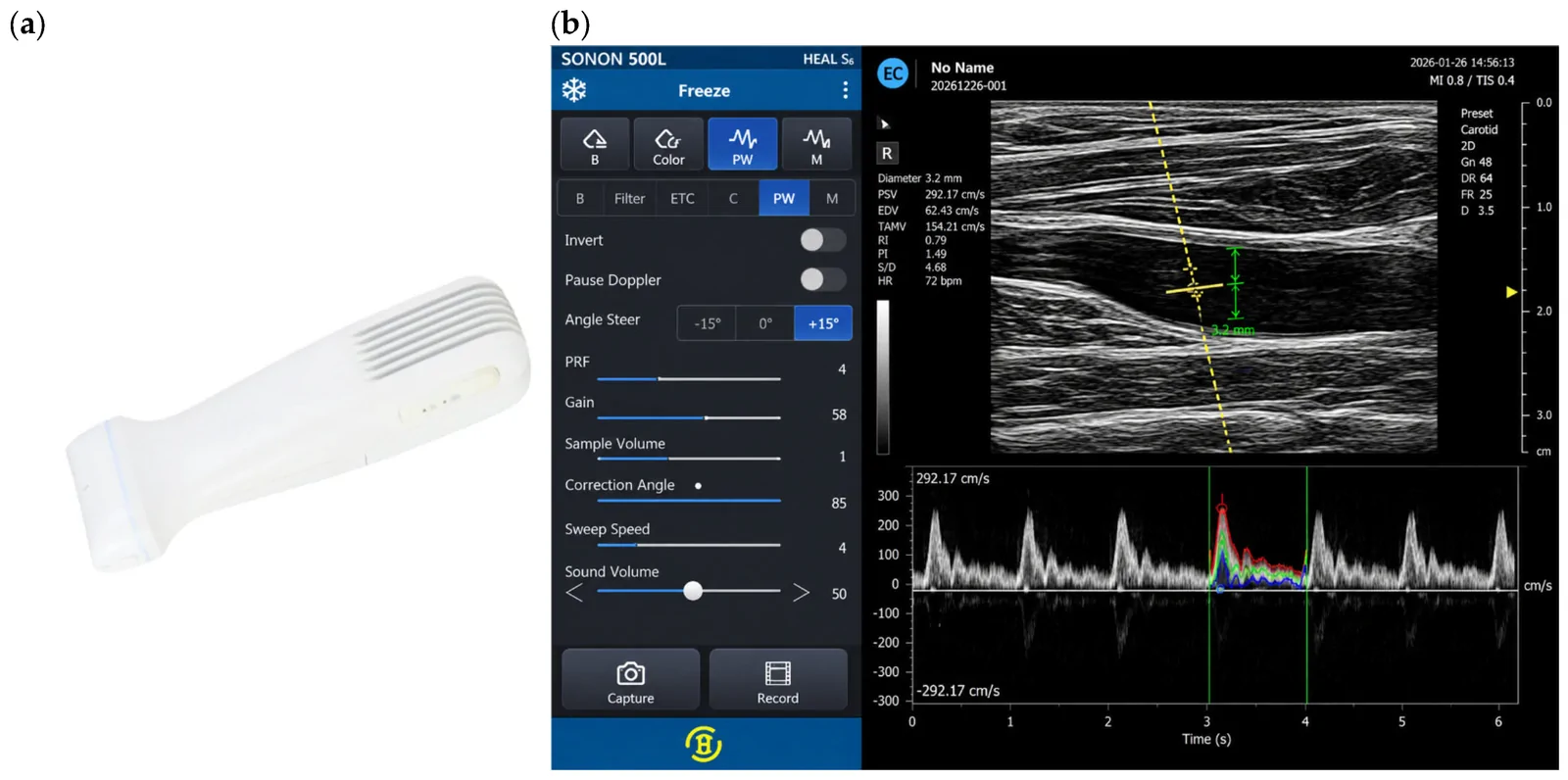
Handheld point-of-care ultrasound device and imaging interface. (**a**) SONON 500L wireless handheld ultrasound device (HEALCERION Co., Ltd., Seoul, Republic of Korea) used for point-of-care ultrasonography. (**b**) Representative ultrasound imaging interface obtained using the SONON 500L system, showing B-mode imaging and pulsed-wave Doppler assessment. The images are reproduced with appropriate permission obtained by the authors.

For Doppler assessment of distal circulation after tourniquet application, a wireless handheld ultrasound device (SONON 500L; HEALCERION Co., Ltd., Seoul, Republic of Korea) was used. The device is a compact, mobile-based point-of-care ultrasound system that provides real-time imaging through wireless connectivity to a mobile device. It supports B-mode imaging and pulsed-wave (PW) Doppler, enabling anatomical identification of the target vessel and simultaneous assessment of blood-flow signals (Figure 3). The handheld and wireless configuration allows rapid bedside assessment without requiring a conventional cart-based ultrasound system and facilitates repeated measurements during tourniquet application. In this study, distal blood-flow signals were evaluated before and after tourniquet application to assess changes in peripheral perfusion. After the tourniquet was applied to the upper extremity under the standardized protocol, Doppler mode was used to evaluate distal blood flow distal to the tourniquet. The presence or absence of a distal flow signal was recorded as a binary outcome (Yes/No). The same ultrasound system was used for both the participant-performed Doppler assessment and the independent assessor’s Doppler confirmation.

### 2.5. Statistical Analysis

Baseline characteristics and procedure-related variables were analyzed using descriptive statistics. After assessing normality, continuous variables were presented as means and standard deviations when the normality assumption was met, and categorical variables were presented as frequencies and percentages.

The primary outcome was the agreement of manual pulse palpation and participant-performed Doppler ultrasonography with assessor-performed Doppler ultrasonography as the reference assessment. For each index test, sensitivity, specificity, overall accuracy, positive predictive value (PPV), and negative predictive value (NPV) were calculated relative to the reference assessment, with 95% confidence intervals (CIs) estimated using the exact binomial method.

Secondary analyses included agreement between the two index tests, paired comparisons of binary classifications, paired comparison of classification accuracy relative to the reference assessment, differences in assessment time, and exploratory subgroup analyses according to field experience and training frequency. Agreement between manual pulse palpation and participant-performed Doppler ultrasonography was evaluated using overall percent agreement and Cohen’s kappa coefficient, with corresponding 95% CIs. Differences in the marginal proportions of “flow present” classifications between the two index tests were evaluated using McNemar’s test based on discordant Yes/No pairs. To directly compare classification accuracy relative to the assessor-performed Doppler reference assessment, each index-test result was classified as correct or incorrect for each participant. A paired 2 × 2 table of correct and incorrect classifications was constructed. The paired difference in accuracy was calculated as the accuracy of participant-performed Doppler ultrasonography minus that of pulse palpation and was reported in percentage points with a 95% CI. Because both index tests were performed in the same participants, the difference in correct classification between the two methods was evaluated using McNemar’s test based on discordant correct/incorrect pairs. Assessment time was analyzed using a paired *t*-test when the distribution of paired differences approximated normality; otherwise, the Wilcoxon signed-rank test was used. Subgroup analyses according to field experience and tourniquet training/practice frequency were considered exploratory descriptive analyses because of the small subgroup sample sizes. No formal interaction tests were performed; therefore, these analyses were not used to infer differential performance between subgroups. All statistical analyses were performed using IBM SPSS Statistics version 25.0. All tests were two-sided, and statistical significance was defined as a *p*-value < 0.05. Because complete paired data were available for all randomized participants, no imputation was performed.

## 3. Results

### 3.1. General Characteristics

The general characteristics of the study participants are presented in Table 1. A total of 41 participants were included, and the sex distribution was similar, with 21 males (51.2%) and 20 females (48.8%). The mean age was 36.4 ± 18.2 years. Regarding field experience, 20 participants (48.8%) had <5 years of experience and 21 participants (51.2%) had ≥5 years. Eighteen participants (43.9%) reported prior experience applying a tourniquet to real patients. The frequency of tourniquet training was <2 times per year in 14 participants (34.1%), 2–5 times per year in 17 participants (41.5%), and ≥6 times per year in 10 participants (24.4%) (Table 1).

### 3.2. Procedure-Related Variables and Assessment Time in the Randomized Crossover Design

Table 2 summarizes the procedure-related variables and assessment times according to the randomized assessment sequence. Of the 41 participants, 21 (51.2%) were assigned to Sequence A (pulse palpation followed by participant-performed Doppler ultrasonography), and 20 (48.8%) were assigned to Sequence B (participant-performed Doppler ultrasonography followed by pulse palpation). Tourniquet application duration was standardized at 2 min for all participants. According to the reference standard (assessor-performed Doppler ultrasonography), distal flow was present in 18 participants (43.9%) and absent in 23 (56.1%). Pulse palpation identified distal flow as present in 15 participants (36.6%) and absent in 26 (63.4%), whereas participant-performed Doppler ultrasonography identified distal flow as present in 18 participants (43.9%) and absent in 23 (56.1%).

The mean assessment time was 14.2 ± 6.1 s for pulse palpation and 28.5 ± 10.4 s for participant-performed Doppler ultrasonography. The mean paired difference in assessment time (Doppler − palpation) was 14.3 ± 9.8 s (95% CI, 11.2–17.4), indicating that participant-performed Doppler ultrasonography required significantly more time than pulse palpation (paired *t*-test, *p* < 0.001). When examined according to the randomized assessment sequence, the mean pulse palpation times were 14.8 ± 6.3 s in Sequence A and 13.5 ± 5.9 s in Sequence B, while the corresponding participant-performed Doppler assessment times were 29.1 ± 10.8 s and 27.8 ± 10.0 s, respectively. The mean paired time difference was 14.3 s in both sequences (Sequence A, 14.3 ± 10.1 s; Sequence B, 14.3 ± 9.6 s) (Table 2).

Doppler assessment time was similar between the two randomized sequences (29.1 ± 10.8 s in Sequence A and 27.8 ± 10.0 s in Sequence B), suggesting no substantial sequence-related difference in assessment time. However, because each participant performed the study assessment within a single crossover session rather than through serial repeated Doppler assessments, a formal learning-curve analysis was not possible. Therefore, the present data should not be interpreted as demonstrating the presence or absence of a learning effect.

### 3.3. Performance of the Index Tests Relative to the Reference Doppler Assessment

Table 3 presents the diagnostic performance of pulse palpation and participant-performed Doppler ultrasonography, using assessor-performed Doppler ultrasonography as the reference standard. For pulse palpation, the sensitivity was 66.7% (95% CI, 41.0–86.7), the specificity was 87.0% (95% CI, 66.4–97.2), and the accuracy was 78.0% (95% CI, 62.4–89.4). The positive predictive value (PPV) was 80.0% (95% CI, 51.9–95.7), and the negative predictive value (NPV) was 76.9% (95% CI, 56.4–91.0). In contrast, participant-performed Doppler ultrasonography showed overall higher performance than pulse palpation, with a sensitivity of 88.9% (95% CI, 65.3–98.6), a specificity of 91.3% (95% CI, 72.0–98.9), and an accuracy of 90.2% (95% CI, 76.9–97.3). The PPV and NPV for participant-performed Doppler ultrasonography were 88.9% (95% CI, 65.3–98.6) and 91.3% (95% CI, 72.0–98.9), respectively (Table 3).

### 3.4. Paired Comparison of Classification Accuracy Between Pulse Palpation and Participant-Performed Doppler Ultrasonography

Relative to the assessor-performed Doppler reference assessment, pulse palpation correctly classified 32 of 41 participants (78.0%), whereas participant-performed Doppler ultrasonography correctly classified 37 of 41 participants (90.2%). In the paired analysis, 30 participants were correctly classified by both methods, 7 were correctly classified by participant-performed Doppler ultrasonography but incorrectly classified by pulse palpation, 2 were correctly classified by pulse palpation but incorrectly classified by participant-performed Doppler ultrasonography, and 2 were incorrectly classified by both methods. The paired accuracy difference between participant-performed Doppler ultrasonography and pulse palpation was 12.2 percentage points (95% CI, −1.7 to 26.0). McNemar’s exact test based on the discordant correct/incorrect pairs showed no statistically significant difference in classification accuracy between the two methods (*p* = 0.180) (Table 4).

### 3.5. Agreement and Paired Comparison Between Pulse Palpation and Participant Doppler Ultrasonography

Table 5 presents the agreement between pulse palpation and participant-performed Doppler ultrasonography and the paired comparison results. In the cross-classification of test results, 13 participants showed concordant “Yes” results for both pulse palpation and participant Doppler, and 21 participants showed concordant “No” results for both methods. Discordant pairs were observed in 2 participants with pulse palpation “Yes”/Doppler “No” and in 5 participants with pulse palpation “No”/Doppler “Yes” (Table 5).

The overall percent agreement was 82.9% (95% CI, 68.1–93.2), and Cohen’s kappa was 0.65 (95% CI, 0.41–0.88), indicating substantial agreement. McNemar’s exact test based on discordant pairs showed no statistically significant difference in the proportion of positive classifications between the two methods (*p* = 0.453) (Table 6).

### 3.6. Exploratory Subgroup Analysis by Field Experience

Table 7 presents the diagnostic performance of pulse palpation and participant-performed Doppler ultrasonography stratified by field experience (<5 years vs. ≥5 years). In the <5 years group (*n* = 20), pulse palpation showed a sensitivity of 60.0% (95% CI, 25.9–88.0) and a specificity of 80.0% (95% CI, 44.4–97.5), whereas participant-performed Doppler ultrasonography showed a sensitivity of 90.0% (95% CI, 55.5–99.7) and a specificity of 90.0% (95% CI, 55.5–99.7). In the ≥5 years group (*n* = 21), the sensitivity and specificity of pulse palpation were 75.0% (95% CI, 34.9–96.8) and 92.3% (95% CI, 64.0–99.8), respectively, and those of participant-performed Doppler ultrasonography were 87.5% (95% CI, 47.3–99.7) and 92.3% (95% CI, 64.0–99.8), respectively (Table 6).

### 3.7. Exploratory Subgroup Analysis by Tourniquet Training/Practice Frequency

Table 8 presents the diagnostic performance of pulse palpation and participant-performed Doppler ultrasonography stratified by tourniquet practice frequency (<2, 2–5, and ≥6 times/year). In the <2 times/year group (*n* = 14), pulse palpation showed a sensitivity of 50.0% (95% CI, 11.8–88.2) and a specificity of 87.5% (95% CI, 47.3–99.7), while participant-performed Doppler ultrasonography showed a sensitivity of 83.3% (95% CI, 35.9–99.6) and a specificity of 87.5% (95% CI, 47.3–99.7). In the 2–5 times/year group (*n* = 17), pulse palpation demonstrated a sensitivity of 75.0% (95% CI, 34.9–96.8) and a specificity of 88.9% (95% CI, 51.8–99.7), whereas participant-performed Doppler ultrasonography showed a sensitivity of 87.5% (95% CI, 47.3–99.7) and a specificity of 88.9% (95% CI, 51.8–99.7). In the ≥6 times/year group (*n* = 10), pulse palpation had a sensitivity of 75.0% (95% CI, 19.4–99.4) and a specificity of 83.3% (95% CI, 35.9–99.6); participant-performed Doppler ultrasonography achieved a sensitivity of 100.0% (95% CI, 39.8–100.0) and a specificity of 100.0% (95% CI, 54.1–100.0) (Table 7).

## 4. Discussion

This randomized crossover study compared pulse palpation and paramedic-performed Doppler ultrasonography for assessing distal circulation after tourniquet application, using assessor-performed Doppler ultrasonography as the reference assessment. Relative to the reference assessment, paramedic-performed Doppler ultrasonography showed a numerically higher sensitivity (88.9% vs. 66.7%) and accuracy (90.2% vs. 78.0%) than pulse palpation. However, the paired analysis of correct and incorrect classifications showed an accuracy difference of 12.2 percentage points (95% CI, −1.7 to 26.0), which was not statistically significant (McNemar’s exact test, *p* = 0.180). Therefore, although participant-performed Doppler showed greater numerical concordance with the assessor-performed Doppler reference assessment, the present data do not establish a statistically significant difference in classification accuracy between the two methods. Furthermore, because participant-performed and assessor-performed Doppler assessments used the same imaging modality, these findings should be interpreted as agreement with the reference Doppler assessment rather than evidence of definitive diagnostic superiority. Pulse palpation may be affected by examiner proficiency and by environmental or physiological factors such as glove use, noise, vibration, low-light conditions, hypothermia, and reduced peripheral perfusion [12,13]. In this context, handheld Doppler ultrasonography may provide an additional objective method for assessing residual distal flow after tourniquet application. However, given the absence of a statistically significant paired difference in classification accuracy and the use of assessor-performed Doppler as the reference assessment, the present findings should be regarded as preliminary and should not be interpreted as demonstrating clinical superiority of Doppler over pulse palpation.

Similar prior studies have repeatedly raised concerns that confirming tourniquet effectiveness, defined as distal arterial flow occlusion, is difficult when relying solely on the loss of a distal pulse by palpation. Multiple studies have verified tourniquet success by confirming the disappearance of distal flow signals using Doppler ultrasound, and they have also noted potential discordance and limitations of palpation and simplified device-based methods such as pulse oximetry [14,15]. Against this background, the present study is meaningful because it directly compared pulse palpation with Doppler assessments performed by paramedics while using assessor-performed Doppler ultrasonography as the reference standard. Previous studies of prehospital POCUS have demonstrated that appropriately trained emergency medical technicians and paramedics can acquire clinically interpretable ultrasound images in selected applications [16,17]. However, these studies have primarily evaluated thoracic or general prehospital POCUS rather than peripheral vascular Doppler assessment following tourniquet application. Therefore, their findings provide contextual evidence for the feasibility of paramedic-performed ultrasonography but should not be considered direct evidence for the specific Doppler application evaluated in the present study. Consistent with the feasibility of ultrasound assessment by non-physician prehospital providers, participant-performed Doppler ultrasonography in the present study showed substantial agreement with the assessor-performed Doppler reference assessment. Although its observed accuracy was numerically higher than that of pulse palpation, the paired difference in classification accuracy was not statistically significant and should therefore be interpreted cautiously. At the same time, Doppler assessment involves a practical trade-off because it requires more time than palpation, with an average additional assessment time of approximately 14 s in the present study.

Therefore, rather than fully replacing palpation, a stepwise approach may be more appropriate in practice. Rapid initial assessment can be performed by palpation, followed by Doppler ultrasonography when palpation findings are equivocal or when objective reassessment is required. In this context, Doppler may be particularly useful as an adjunctive confirmation tool immediately after tourniquet placement and during subsequent reassessment before or during transport. Nevertheless, several practical challenges should be considered when translating handheld Doppler assessment into prehospital trauma care. Unlike the controlled environment of the present study, field assessments may be performed under substantial noise, vibration, limited lighting, restricted working space, patient movement, and time pressure. Personal protective equipment and gloves may further complicate device handling and precise probe positioning. In addition, Doppler assessment requires preparation of the device, identification of the target vessel, appropriate probe positioning, and interpretation of the flow signal. These operational factors could increase assessment time or reduce measurement reliability in real-world settings. Therefore, the diagnostic performance observed under controlled conditions should not be assumed to translate directly to all prehospital environments. The additional time required for Doppler assessment should also be considered in the context of time-critical hemorrhage control. Although Doppler assessment required approximately 14 s longer than pulse palpation in the present study, our study was not designed to determine whether this additional time translates into clinically meaningful treatment delay or affects patient outcomes. Importantly, Doppler assessment should not delay immediate tourniquet application or other life-saving hemorrhage-control interventions. Its potential role may therefore be more appropriate as an adjunctive confirmation or reassessment tool after initial hemorrhage control has been achieved, particularly when palpation findings are equivocal. Further field studies are needed to determine whether the additional assessment time remains acceptable under real-world time-critical conditions. More broadly, systematic and scoping reviews of prehospital POCUS suggest that ultrasound is feasible in prehospital critical care and may provide additional diagnostic information, although evidence regarding its effects on patient-centered outcomes remains limited [18,19].

Interpreting these findings within the context of the Republic of Korea’s 119 emergency medical services system further clarifies the potential practical relevance of Doppler implementation. Korea’s prehospital emergency medical system operates as a 119-centered public service, and prehospital interventions are performed within defined legal and institutional frameworks [20,21,22]. Although Doppler ultrasonography is not currently established as a routine assessment method for Korean paramedics, international experience suggests that implementation of prehospital ultrasound requires consideration not only of device availability but also of provider training, competency maintenance, quality assurance, and integration into existing EMS workflows [18,23].

These considerations are particularly relevant in hemorrhage and trauma situations in which conventional pulse palpation may be difficult because of environmental or physiological factors. Under such conditions, Doppler ultrasonography could potentially serve as an adjunctive assessment tool for objectively confirming distal arterial flow after tourniquet application and supporting repeated reassessment. However, its introduction into routine prehospital practice should be accompanied by clearly defined indications, standardized protocols, and appropriate clinical oversight. Previous literature on paramedic-performed tele-ultrasound has also explored models in which prehospital providers acquire ultrasound images with remote expert support, suggesting a potential framework for integrating ultrasound with medical direction systems [24].

Accordingly, if Doppler ultrasonography is considered for implementation in the Korean prehospital setting, its role could initially be limited to a clearly defined purpose, such as confirming tourniquet effectiveness, identifying residual distal flow that may indicate incomplete occlusion, and supporting reassessment during transport. Evidence from the broader prehospital ultrasound literature suggests that such implementation should be evaluated through structured training programs and prospective field studies before routine adoption [18,19].

Exploratory subgroup analyses according to field experience and tourniquet training/practice frequency showed numerical variation in performance estimates; however, these estimates were imprecise, with wide confidence intervals due to the small subgroup sizes. Because no formal interaction analyses were performed, the present findings cannot establish whether the performance of Doppler ultrasonography differs according to provider experience or training frequency. Therefore, these subgroup findings should be considered hypothesis-generating rather than confirmatory. Future studies with larger samples and prespecified interaction analyses are needed to determine whether provider experience or training modifies Doppler assessment performance. Although these subgroup findings are exploratory because of the small sample sizes, they reinforce the importance of education and competency when introducing new assessment technologies into prehospital practice. Previous literature has similarly emphasized structured education, skill maintenance, and quality assurance as important components of prehospital POCUS implementation [23]. Moreover, recent evidence from an EMS system in which POCUS had not yet been routinely implemented identified limited training opportunities, equipment availability, and unclear regulatory frameworks and scopes of practice as important barriers to implementation [25]. Therefore, future implementation strategies should consider Doppler ultrasonography not as a stand-alone device intervention but as part of an integrated program incorporating standardized training, competency assessment, medical oversight, and continuous quality improvement.

Overall, the present study provides preliminary evidence that handheld Doppler ultrasonography may improve the objectivity of distal circulation assessment after tourniquet application compared with pulse palpation alone. In the longer term, these findings support further investigation of paramedic-performed Doppler ultrasonography in real-world prehospital environments and evaluation of the educational, operational, and regulatory requirements necessary for its safe implementation.

The limitations of this study can be summarized in three points. First, the study was conducted in a controlled environment; therefore, real-world transport conditions such as noise, vibration, lighting, and weather were not fully represented, which may limit external validity. In addition, although participant-performed Doppler ultrasonography showed a numerically higher classification accuracy than pulse palpation, the paired accuracy comparison did not reach statistical significance, and the confidence interval around the accuracy difference was wide. Therefore, the study may not have had sufficient precision to establish a difference in classification accuracy between the two methods. Second, the overall sample size was modest, and subgroup analyses according to field experience and training frequency involved small strata with wide confidence intervals; therefore, these findings should be interpreted as exploratory. Third, assessor-performed Doppler ultrasonography was used as the reference standard rather than an independent physiological gold standard. Because participant-performed Doppler ultrasonography and the reference assessment were based on the same imaging modality, some degree of incorporation or modality-related bias cannot be excluded. Therefore, the observed diagnostic performance of participant-performed Doppler ultrasonography should be interpreted as agreement with an expert Doppler assessment rather than definitive diagnostic accuracy against an absolute gold standard. In addition, the reference Doppler assessment was performed immediately after completion of both index tests rather than simultaneously with each index test. Although the interval was minimized and the tourniquet remained in place throughout the assessments, temporal changes in distal arterial flow cannot be completely excluded.

## 5. Conclusions

This randomized crossover study compared pulse palpation with paramedic-performed Doppler ultrasonography for assessing distal circulation after tourniquet application, using assessor-performed Doppler ultrasonography as the reference assessment. Paramedic-performed Doppler ultrasonography showed greater concordance with the reference Doppler assessment than pulse palpation. However, because both the participant-performed and reference assessments used the same Doppler modality, these findings should be interpreted as reflecting agreement with the assessor-performed Doppler assessment rather than definitive diagnostic superiority. In addition, Doppler assessment required more time than palpation; therefore, a stepwise approach may be more appropriate in the field, with rapid initial assessment by palpation followed by Doppler ultrasonography when palpation findings are equivocal or when objective reassessment of distal flow is required before or during transport. These findings suggest that handheld Doppler ultrasonography may have a potential role as an adjunct to pulse palpation for confirming distal flow status after tourniquet application. Within the Republic of Korea’s 119 EMS context, any potential implementation should be considered in relation to existing scopes of practice, provider training, competency maintenance, and medical oversight. Further studies using an independent physiological reference standard and conducted in patients and real-world prehospital environments are warranted to determine the clinical utility, feasibility, and safety of paramedic-performed Doppler ultrasonography.

## Figures and Tables

**Table 1:** Participant characteristics (*N* = 41).

Characteristic	Participants (*N* = 41)
Gender	
Male, *n* (%)	21 (51.2)
Female, *n* (%)	20 (48.8)
Age, years, Mean ± SD	36.4 ± 18.2
Field experience	
<5 years, *n* (%)	20 (48.8)
≥5 years, *n* (%)	21 (51.2)
Prior real-patient tourniquet application experience/yes, *n* (%)	18 (43.9)
Tourniquet practice frequency	
<2 times/year, *n* (%)	14 (34.1)
2–5 times/year, *n* (%)	17 (41.5)
≥6 times/year, *n* (%)	10 (24.4)

Variables were presented as *N* (%) or Mean and standard deviation.

**Table 2:** Procedure-related variables in the randomized crossover design (*N* = 41).

Variables	Total (*N* = 41)	Sequence A: Palpation → Doppler (*n* = 21)	Sequence B: Doppler → Palpation (*n* = 20)
Reference test (Assessor Doppler) result			
Distal flow present/Yes, *n* (%)	18 (43.9)	9 (42.9)	9 (45.0)
Distal flow absent/No, *n* (%)	23 (56.1)	12 (57.1)	11 (55.0)
Index test result (binary recording structure)			
Pulse palpation/Yes, *n* (%)	15 (36.6)	8 (38.1)	7 (35.0)
Pulse palpation/No, *n* (%)	26 (63.4)	13 (61.9)	13 (65.0)
Participant Doppler/Yes, *n* (%)	18 (43.9)	10 (47.6)	8 (40.0)
Participant Doppler/No, *n* (%)	23 (56.1)	11 (52.4)	12 (60.0)
Assessment time			
Pulse palpation time, sec	14.2 ± 6.1	14.8 ± 6.3	13.5 ± 5.9
Participant Doppler time, sec	28.5 ± 10.4	29.1 ± 10.8	27.8 ± 10.0
Paired difference (Doppler − Palpation)	14.3 ± 9.8	14.3 ± 10.1	14.3 ± 9.6
95% CI for paired difference	11.2–17.4	-	-
Paired comparison, *p*-value	<0.001	-	-

Values are presented as *n* (%) or mean ± standard deviation (SD), as appropriate. Participants were randomized to Sequence A (pulse palpation followed by participant-performed Doppler ultrasonography) or Sequence B (participant-performed Doppler ultrasonography followed by pulse palpation). Assessor-performed Doppler ultrasonography was used as the reference standard. Assessment time was measured in seconds. The paired difference was calculated as participant-performed Doppler assessment time minus pulse palpation assessment time. The 95% confidence interval (CI) and *p*-value for the overall paired difference were calculated using a paired *t*-test.

**Table 3:** Diagnostic performance of index tests versus the reference standard (*N* = 41).

Index Test	TP	FP	FN	TN	Sensitivity %, (95% CI)	Specificity %, (95% CI)	Accuracy %, (95% CI)	PPV %, (95% CI)	NPV %, (95% CI)
Pulse palpation	12	3	6	20	66.7 (41.0–86.7)	87.0 (66.4–97.2)	78.0 (62.4–89.4)	80.0 (51.9–95.7)	76.9 (56.4–91.0)
Doppler ultrasonography	16	2	2	21	88.9 (65.3–98.6)	91.3 (72.0–98.9)	90.2 (76.9–97.3)	88.9 (65.3–98.6)	91.3 (72.0–98.9)

The assessor-performed Doppler ultrasonography served as the reference standard. For each index test, diagnostic accuracy was summarized using true positives (TP), false positives (FP), false negatives (FN), and true negatives (TN). Sensitivity, specificity, accuracy, positive predictive value (PPV), and negative predictive value (NPV) are reported as percentages with 95% confidence intervals (CIs). The 95% CIs were calculated using the exact binomial (Clopper–Pearson) method. TP and TN were defined as concordant results with the reference standard for positive and negative cases, respectively.

**Table 4:** Paired comparison of correct and incorrect classifications between pulse palpation and participant-performed Doppler ultrasonography relative to the assessor-performed Doppler reference assessment (*N* = 41).

Pulse Palpation Classification	Participant Doppler: Correct	Participant Doppler: Incorrect	Total
Correct	30	2	32
Incorrect	7	2	9
Total	37	4	41
Pulse palpation accuracy, *n*/*N* (%)			32/41 (78.0)
Participant Doppler accuracy, *n*/*N* (%)			37/41 (90.2)
Paired accuracy difference, percentage points			12.2
95% CI for paired accuracy difference			−1.7 to 26.0
McNemar’s exact test, *p*-value			0.180

Correct classification was defined as agreement between the index-test result and the assessor-performed Doppler reference assessment, whereas incorrect classification was defined as disagreement with the reference assessment. The paired accuracy difference was calculated as the accuracy of participant-performed Doppler ultrasonography minus that of pulse palpation and is expressed in percentage points. The 95% confidence interval (CI) for the paired accuracy difference was calculated based on the paired binary data. McNemar’s exact test was performed using discordant correct/incorrect pairs to compare classification accuracy between the two index tests. All analyses were based on complete paired data from 41 participants. CI, confidence interval.

**Table 5:** Paired comparison and agreement between the two index tests (2 × 2 paired table, Pulse palpation vs. Participant Doppler).

Variables	Participant Doppler (Yes)	Participant Doppler (No)	Total
Pulse palpation: Yes	13	2	15
Pulse palpation: No	5	21	26
Total	18	23	41

This table presents the paired cross-classification of the two index tests within the same participants (*N* = 41): pulse palpation versus participant Doppler ultrasonography. Cells represent the number of participants with each combination of binary results (Yes/No). The off-diagonal cells indicate discordant pairs (palpation Yes/Doppler No = 2; palpation No/Doppler Yes = 5) and were used for the McNemar test. Overall agreement and chance-corrected agreement were quantified using percent agreement and Cohen’s kappa, respectively (reported with 95% CI).

**Table 6:** Agreement and paired comparison statistics.

Measure	Value
Overall percent agreement, % (95% CI)	82.9 (68.1–93.2)
Cohen’s kappa (95% CI)	0.65 (0.41–0.88)
McNemar’s exact test (discordant pairs: 2 vs. 5), *p*-value	0.453

Overall percent agreement was calculated as (a+d)/N, where a and d are the concordant cells in the paired 2 × 2 table. The 95% CI for percent agreement was obtained using an exact binomial method. Cohen’s kappa was used to quantify chance-corrected agreement, with its 95% CI estimated using standard large-sample methods. McNemar’s exact test was performed on the discordant pairs (b vs. c) to compare marginal proportions between pulse palpation and participant Doppler ultrasonography; two-sided *p*-values are reported.

**Table 7:** Exploratory subgroup analysis of pulse palpation and participant-performed Doppler ultrasonography according to field experience.

Subgroup	Index Test	TP	FP	FN	TN	Sensitivity % (95% CI)	Specificity % (95% CI)
<5 years (*n* = 20)	Pulse palpation	6	2	4	8	60.0 (25.9–88.0)	80.0 (44.4–97.5)
	Participant Doppler	9	1	1	9	90.0 (55.5–99.7)	90.0 (55.5–99.7)
≥5 years (*n* = 21)	Pulse palpation	6	1	2	12	75.0 (34.9–96.8)	92.3 (64.0–99.8)
	Participant Doppler	7	1	1	12	87.5 (47.3–99.7)	92.3 (64.0–99.8)

Subgroup analyses were stratified by field experience (<5 years vs. ≥5 years). Within each subgroup, diagnostic performance of each index test (pulse palpation and participant Doppler ultrasonography) was evaluated against the reference standard (assessor-performed Doppler ultrasonography). Sensitivity and specificity are presented as percentages with 95% confidence intervals (CIs), calculated using the exact binomial (Clopper–Pearson) method. The subgroup sample size (n) denotes the number of participants included in that stratum. Results are descriptive and should be interpreted cautiously due to the small subgroup sample sizes.

**Table 8:** Exploratory subgroup analysis of pulse palpation and participant-performed Doppler ultrasonography according to tourniquet training/practice frequency.

Subgroup	Index Test	TP	FP	FN	TN	Sensitivity %, (95% CI)	Specificity %, (95% CI)
<2 times/year (*n* = 14)	Pulse palpation	3	1	3	7	50.0 (11.8–88.2)	87.5 (47.3–99.7)
	Participant Doppler	5	1	1	7	83.3 (35.9–99.6)	87.5 (47.3–99.7)
2–5 times/year (*n* = 17)	Pulse palpation	6	1	2	8	75.0 (34.9–96.8)	88.9 (51.8–99.7)
	Participant Doppler	7	1	1	8	87.5 (47.3–99.7)	88.9 (51.8–99.7)
≥6 times/year (*n* = 10)	Pulse palpation	3	1	1	5	75.0 (19.4–99.4)	83.3 (35.9–99.6)
	Participant Doppler	4	0	0	6	100.0 (39.8–100.0)	100.0 (54.1–100.0)

Subgroup analyses were stratified by tourniquet practice frequency (<2, 2–5, and ≥6 times/year). Within each stratum, diagnostic performance of each index test (pulse palpation and participant Doppler ultrasonography) was assessed against the reference standard (assessor-performed Doppler ultrasonography). Sensitivity and specificity are reported as percentages with 95% confidence intervals (CIs), calculated using the exact binomial (Clopper–Pearson) method. The subgroup sample size (n) indicates the number of participants within each practice-frequency category. Given the small stratum sizes, these results are intended as descriptive, supportive analyses and should be interpreted with caution.

## Data Availability

The data supporting the findings of this study are available from the corresponding author upon reasonable request. The data are not publicly available due to privacy and ethical restrictions related to the protection of participant information.

## Abbreviations

The following abbreviations are used in this manuscript:

ED	Emergency department
EMS	Emergency medical services
IRB	Institutional Review Board
ATLS	Advanced Trauma Life Support
xABCDE	eXsanguinating, Airway, Breathing, Circulation, Disability, Exposure
CAT	Combat Application Tourniquet
PHTLS	Prehospital Trauma Life Support
NAEMT	National Association of Emergency Medical Technicians
